# Beneficial Effects of Probiotic and Food Borne Yeasts on Human Health 

**DOI:** 10.3390/nu2040449

**Published:** 2010-04-01

**Authors:** Saloomeh Moslehi-Jenabian, Line Lindegaard Pedersen, Lene Jespersen

**Affiliations:** Department of Food Science, Food Microbiology, Faculty of Life Sciences, University of Copenhagen, Rolighedsvej 30, DK-1958 Frederiksberg C, Denmark; Email: smje@life.ku.dk (S.M.J.); llp@life.ku.dk (L.L.P.)

**Keywords:** yeasts, *S. cerevisiae* var. *boulardii*, probiotics, phytate, folate, mycotoxins

## Abstract

Besides being important in the fermentation of foods and beverages, yeasts have shown numerous beneficial effects on human health. Among these, probiotic effects are the most well known health effects including prevention and treatment of intestinal diseases and immunomodulatory effects. Other beneficial functions of yeasts are improvement of bioavailability of minerals through the hydrolysis of phytate, folate biofortification and detoxification of mycotoxins due to surface binding to the yeast cell wall.

## 1. Introduction

Fermentation is one of the oldest forms of food processing and preservation in the world. Since very early times, humans have been exploiting yeasts and their metabolic products, mainly for baking and brewing. Nowadays, the products of modern yeast biotechnology form the backbone of many commercially important sectors, including foods, beverages, pharmaceuticals, industrial enzymes and others. *Saccharomyces cerevisiae*, which according to EFSA (The European Food Safety Authority) has a QPS (Qualified Presumption of Safety) status [1], is the most common yeast used in food fermentation where it has shown various technological properties. Yeasts do also play a significant role in the spontaneous fermentation of many indigenous food products. A review on *S. cerevisiae* in African fermented foods has been provided by Jespersen [2]. Several beneficial effects on human health and well-being have been reported and there seems to be a need to understand the positive effects of yeasts, their mechanisms and employment of them. The present article reviews the major beneficial effects of yeasts, *i.e.,* probiotic effects, biodegradation of phytate, folate biofortification and detoxification of mycotoxins, which has been summarized in Table 1. However, there are other reported effects such as enrichment of foods with prebiotics as fructooligosaccharides [3], lowering of serum cholesterol [4,5], antioxidative properties, antimutagenic and antitumor activities [6] etc. These topics will meanwhile not be the focus of the present review. Additional information on health significance and food safety of yeasts in foods and beverages can be obtained from Fleet and Balia [7].

**Table 1:** Overview of the major beneficial effects of yeasts.

Activity	Yeast species	Heath effects	Ref.
Probiotic effect	*Saccharomyces cerevisiae* var. *boulardii*	Effect on enteric bacterial pathogen	[16,17,18,19,20,21,22,23,24,25,26,27,28,29,30,31,32,33,34,35]
		Maintenance of epithelial barrier integrity	[21,22,31,36]
		Anti-inflammatory effects	[21,22,31,32,33,34,35,37,39,40,41]
		Effects on immune response	[42,43,44,45]
		Trophic effects on intestinal mucosa	[46,47,48,49,52,53]
		Clinical effects on diarrheal diseases	[62,63,65,66,67,68,69,70,71,72,73,74,75]
Biodegradation of phytate	*Saccharomyces cerevisiae*, *Saccharomyceskluyveri, Schwanniomyces castellii,Debaryomyces castellii*, *Arxula adeninivorans,Pichia anomala*, *Pichia rhodanensis, Pichia spartinae*, *Cryptococcus laurentii*, *Rhodotorula gracilis*, *Torulaspora delbrueckii, Kluyveromyces lactis Candida krusei* (*Issatchenkia orientalis*) and *Candida* spp.	Nutritional importance, *i.e.*, bioavailability of divalent minerals such as iron, zink, calcium and magnesium	[87,88,89,90,91,92,93,94,95,96,97,98,99,100,101,102,103,104,105,106,107,108]
Folate biofortification	*S. cerevisiae*	Prevention of neural tube defects in the foetus, megaloblastic anaemia and reduction of the risk for cardiovascular disease, cancer and Alzheimer's disease	[109,110,111,112,113,114,115,116,117,118,119,120,121,122,123,124,125,126,130]
	*Saccharomyces bayanus, Saccharomyces paradoxus, Saccharomyces pastorianus, Metschnikowia lochheadii, Debaryomyces melissophilus, Debaryomyces vanrijiae* var. *vanrijiae, Debaryomyces hansenii, Pichia philogaea, Kodamaea anthophila, Wickerhamiella lipophilia, Candida cleridarum* and *Candida drosophilae*		[121]
	*Candida milleri* and *T. delbrueckii*		[126]
	*Saccharomyces exiguous* and *Candida lambica*		[128,129]
	*P. anomala* and *Candida glabrata*		[130]
	*Kluyveromyces marxianus* and *C. krusei* (*I. orientalis*)		[128,130]
Degradation of mycotoxins	*S. cerevisiae*	Antitoxic in some degree	[138,139,140,141]
	*Phaffia rhodozyma* and *Xanthophyllomyces dendrorhous*		[142]
Absorption of mycotoxins	*S. cerevisiae*	Antitoxic	[143,144,145]

## 2. Beneficial Effects of Yeast as Probiotics

### 2.1. Taxonomic Characterization of Probiotic Yeasts

Probiotics are defined as ‘live microorganisms which when administered in adequate amounts confer a health benefit on the host’ [8]. Probiotics may be consumed either as food components or as non-food preparations. There is a great interest in finding yeast strains with probiotic potential. Different yeast species such as *Debaryomyces hansenii, Torulaspora delbrueckii* [9], *Kluyveromyces lactis, Kluyveromyces marxianus, Kluyveromyces lodderae* [10] have shown tolerance to passage through the gastrointestinal tract or inhibition of enteropathogens. However, *Saccharomyces boulardii* is the only yeast with clinical effects and the only yeast preparation with proven probiotic efficiency in double-blind studies [11]. *S. boulardii*, isolated from litchi fruit in Indochina by Henri Boulard in the 1920s, is commonly used as a probiotic yeast especially in the pharmaceutical industry and in a lyophilized form for prevention and treatment of diarrhoea. In a study conducted by van der Aa Kühle and Jespersen [12] on commercial strains of *S. boulardii,* it was found that the *S. boulardii* strains morphologically and physiologically could be characterized as *S. cerevisiae*. Sequences of the D1/D2 domain of the 26S rRNA gene were identical for all isolates examined and had 100 % similarity with the sequences of the type strain of *S. cerevisiae* (CBS 1171^T^) and the sequenced *S. cerevisiae* strain S288c. All *S. boulardii* isolates were found to have the same ITS1-5.8S rRNA-ITS2 sequence, which displayed a close resemblance with the sequences published for S288c (99.9%), CBS 1171^T^ (99.3%) and other *S. cerevisiae* strains. Sequence analysis of the mitochondrial cytochrome-*c* oxidase II gene (*COX2*) also resulted in identical sequences for the *S. boulardii* strains and comparisons with available nucleotide sequences revealed close relatedness to strains of *S. cerevisiae* including S288c (99.5%) and CBS 1171^T^ (96.6%). The electrophoretic karyotypes of the *S. boulardii* strains appeared quite uniform and although very typical of *S. cerevisiae*, they formed a cluster separate from other strains within this species. The results of the study strongly indicated a close relatedness of *S. boulardii* to *S. cerevisiae* and thereby support the recognition of *S. boulardii* as a member of *S. cerevisiae* and not as a separate species. The fact that strains of *S.boulardii* should be seen as a separate cluster within the *S. cerevisiae* species is further supported by the fact that strains of *S. boulardii* previously have been reported to differ from strains of *S. cerevisiae* due to a specific microsatellite allele [13] as well as trisomy of the chromosome IX and altered copy numbers of specific genes [14]. Others have reported *S. boulardii* strains to tolerate acidic stress better and grow faster at 37 °C than *S. cerevisiae* [15]. Due to the fact that *S. boulardii* from a taxonomic point of view should not be recognized as a separate species, *S. boulardii* will in the following be referred to as *S. cerevisiae* var. *boulardii*. It is worth to notice that contrary to e.g., probiotic strains of lactic acid bacteria, apparently there seems not to be different strains within *S. cerevisiae* var. *boulardii.* Based on the similarity in different molecular analyses, all isolates appear to originate from the one isolated from litchi fruit in Indochina by Henri Boulard [12]. 

### 2.2. Experimental Effects of *S. cerevisiae* var. *boulardii*

#### 2.2.1. Effects on enteric bacterial pathogens

Several studies have shown that S. cerevisiae var. boulardii confer beneficial effects against various enteric pathogens, involving different mechanisms as: (i) prevention of bacterial adherence and translocation in the intestinal epithelial cells, (ii) production of factors that neutralize bacterial toxins and (iii) modulation of the host cell signalling pathway associated with pro-inflammatory response during bacterial infection.

Prevention of bacterial adherence and translocation in the intestinal epithelial cells is due to the fact that the cell wall of S. cerevisiae var. boulardii has the ability to bind enteropathogens. S. cerevisiae var. boulardii cell wall has shown binding capacity to enterohaemorrhagic *Escherichia coli* and *Salmonella enterica* serovar Typhimurium [16]. Additionally, the yeast inhibits adherence of *Clostridiumdifficile* to Vero cells (derived from kidney epithelial cells). Pre-treatment of *C. difficile* or the Vero cells with *S. cerevisiae* var. *boulardii* or its cell wall particles results in lowering the adherence of bacteria to the Vero cells. Yeast cells or cell wall particles are able to modify the surface receptors involved in adhesion of *C. difficile* through a proteolytic activity and by steric hindrance [17]. Administration of *S. cerevisiae* var. *boulardii* reduces adherence of enterotoxigenic *E. coli* to mesenteric lymph node in pigs intestine [18]. *S. cerevisiae* var. *boulardii* has also beneficial effect on *Citrobacter* *rodentium*-induced colitis in mice, which is due to attenuating the adherence of *C. rodentium* to host epithelial cells, through reduction in EspB and Tir protein secretions, respectively a translocator and an effector protein implicated in the type III secretion system (TTSS) [19]. In a study on rats, ingestion of *S. cerevisiae* var. *boulardii* decreased the incidence of antibiotic-induced bacterial translocation. The total bacteria count of fecal flora and especially the number of Gram-negative bacteria were significantly lower after intake of the yeast in addition to antibiotic [20]. However, in other studies on enteropathogenic E. coli- or *Shigella*-infected T84 cells (human colonic adenocarcinoma cell line) and on mice infected with *S. enterica* serovar Typhimurium or *Shigella flexneri*, in which *S. cerevisiae* var. *boulardii* demonstrated beneficial effects, no effect on modifying the bacterial adherence was observed [21,22,23].

*S. cerevisiae* var. *boulardii* produces two proteins of 54 and 120 kDa being responsible for degradation or neutralisation of bacterial toxins. The 54 kDa protein is a serine protease that decrease the  enterotoxic and cytotoxic activities of *C. difficile* by proteolysis of the toxin A and inhibition of binding of the toxin to its brush border membrane receptor. *In vivo* studies have shown that oral administration of S. cerevisiae var. boulardii or its supernatant decreases toxin A-induced intestinal secretion and permeability due to activity of this enzyme [24,25,26]. The 120 kDa protein has a non-proteolytic activity, competes specifically with the chloride secretion stimulated by the toxins of *Vibrio cholera* by reducing the cyclic adenosine monophosphate (cAMP) in the intestinal cells [27,28]. Both *S. cerevisiae* var. *boulardii* and *S. cerevisiae* W303 have the ability to protect Fisher rats against cholera toxin [29]. *S. cerevisiae* var. *boulardii* also synthesizes a protein phosphatase that dephosphorylates endotoxins such as lipopolysaccharide of *E. coli* 055B5 and inactivates its cytotoxic effects [30].

*In vitro* studies using mammalian cell cultures have shown that S. cerevisiae var. boulardii modifies host cell signalling pathways associated with pro-inflammatory response during bacterial infection. The mechanism is based on blocking activation of nuclear factor-kappa B (NF-κB) and mitogen-activated protein kinase (MAPK) which decreases the expression of inflammation-associated cytokines such as interleukin 8 (IL-8), tumor necrosis factor alpha (TNF-α) and interferon gamma (IFN-γ) [22,31,32]. The exposure of mammalian cells to S. cerevisiae var. boulardii before addition of enteropathogenic and enterohaemorrhagic E. coli reduces activation of NF-κB and MAPK, diminish production of TNF-α and secretion of IL-8 [21,31], delay enterohaemorrhagic E. coli -induced apoptosis (due to the reduction of TNF-α) and decline pro-inflammatory cytokine synthesis [32]. *S. cerevisiae* var. *boulardii* produces a 10 kDa protein that exerts anti-inflammatory effects after stimulation with *C. difficile*-toxin A due to decrease in secretion of IL-8 in human colonocytes and activation of extracellular signal-regulated protein kinases 1 and 2 (ERK1/2) in both human colonocytes and murine ileal loops [33]. Sougioultzis *et al.* [34] has shown that *S. cerevisiae* var. *boulardii* produces a low molecular weight soluble factor (< 1 kDa) which blocks NF-κB activation and NF-κB-mediated IL-8 gene expression in intestinal epithelial cells and monocytes. Expression of the pro-inflammatory cytokine IL-1α also decreased in IPEC-J2 cells (porcine intestinal epithelial cell lines) exposed to enterohaemorrhagic *E. coli*, when cells were pre- and co-incubated with *S. cerevisiae* var. *boulardii* [35].

2.2.2 Maintenance of epithelial barrier integrity

Klingberg *et al.* [36] have shown that exposure of different strains of *S. cerevisiae* var. *boulardii* and *S. cerevisiae* to Caco-2 cells (human epithelial colorectal adenocarcinoma cell lines) increased the transepithelial electrical resistance (TER) across polarized monolayers of cells. In another study, infection of T84 cells with enteropathogenic *E. coli* reduced the monolayer transepithelial resistance and distribution of tight-junction-associated protein *Zonula occludens* (ZO-1) was altered, which caused disruption of epithelial barrier structure [21]. Presence of *S. cerevisiae* var. *boulardii* in the infection showed no alteration in the transepithelial resistance and ZO-1 protein distribution, suggesting a protective effect of *S. cerevisiae* var. *boulardii* on the tight-junctions structure of T84-infected cells. During bacterial infection, the myosin light chain protein (MLC) is phosphorilated and the tight-junctions are disrupted. Dahan *et al.* [31] have shown that *S. cerevisiae* var. *boulardii* abolished phosphorylation of MLC and thereby eliminated the reduction of TER after infection of cells with enterohaemorrhagic *E. coli* and in that way preserved the barrier function. In *Shigella*-infected T84 cells, the yeast positively affected tight-junctions proteins (claudin-1 and ZO-2) and significantly protected the barrier function [22]. *Shigella*-infected cellular monolayer had a dramatic decrease in claudin-1 and ZO-2 levels. In the presence of yeast, cellular monolayer exhibited larger amounts of these proteins. These results demonstrate that *S. cerevisiae* var. *boulardii* enhances the ability of intestinal epithelial cells to restore the tight-junction structure and the barrier permeability.

#### 2.2.3. Anti-inflammatory effects

Besides reducing inflammation during bacterial infection by interfering with the host cell signalling pathways, *S. cerevisiae* var. *boulardii* also stimulates the peroxisome proliferator-activated receptor-gamma (PPAR-γ) expression in human colonocytes and reduces the response of human colon cells to pro-inflammatory cytokines [37]. PPAR-γ is a nuclear receptor expressed by several cell types including intestinal epithelial cells, dendritic cells, T and B cells, and can act as a regulator of the inflammation [38]. *S. cerevisiae* var. *boulardii* has been reported to modify the migratory behaviour of lymphocytes. This was observed in a mice model of inflammatory bowel disease (IBD), where inhibition of inflammation in the colon was detected in animals treated with *S. cerevisiae* var. *boulardii*. The inhibition was due to decrease in the production of IFN-γ and a modification of T cell distribution. There was a decrease in IFN-γ-producing CD4^+^ T cells within the colonic mucosa and an increase in IFN-γ-producing T cells in the mesenteric lymph nodes. In addition, *S. cerevisiae* var. *boulardii* supernatant modifies the capacity of endothelial cells to adhere to leucocytes, allowing better cell rolling and adhesion [39]. In inflammatory bowel disease (IBD), production of high levels of nitric oxide (NO) and inducible nitric oxide synthase (iNOS) activity is associated with inflammatory effects [40]. The inhibitory effect of *S. cerevisiae* var. *boulardii* on iNOS activity has been investigated by Girard *et al.* [41] in rats with castor oil-induced diarrhoea. Administration of yeast blocked the production of the citrulline (a marker of NO production). The iNOS inhibition by *S. cerevisiae* var. *boulardii* may be beneficial in the treatment of diarrhoea and/or IBD associated with overproduction of NO. 

#### 2.2.4. Effects on immune response

There are several studies indicating the stimulation of the host cell immunity, both innate and adaptive immunity, by *S. cerevisiae* var. *boulardii* in response to pathogen infections. Oral administration of *S. cerevisiae* var. *boulardii* in healthy volunteers revealed several cellular and humoral changes in peripheral blood. This contributes to the activation of the reticuloendothelial and complement system, demonstrating the stimulation of the innate immune system by the yeast [42]. Oral ingestion of *S. cerevisiae* var. *boulardii* stimulated secretion of immune factors, *i.e.*, adaptive immunity. In a study by Buts *et al.* [43], the level of secretory immunoglobulin A (sIgA) increased 57% in the duodenal fluid and the secretory component of immunoglobulins enhanced 69% in villus cells and 80% in crypt cells of rats treated with the high dose of yeast. Application of *S. cerevisiae* var. *boulardii* to mice treated with *C. difficile* toxin A caused a 1.8-fold increase in total sIgA levels and a 4.4-fold increase in specific antitoxin A sIgA levels [44]. In another study, after intravenous administration of *E. coli*, germ-free mice mono-associated with *S. cerevisiae* var. *boulardii* showed higher clearance of the pathogen from the bloodstream compared to germ-free mice, which was correlated with earlier production of IFN-γ and IL-12 in the serum [45].

#### 2.2.5. Trophic effects on intestinal mucosa

Several studies have shown that *S. cerevisiae* var. *boulardii* exerts trophic effects restoring the intestinal homeostasis. Oral administration of yeast by human volunteers or rats enhanced the activity of brush border membrane enzymes, e.g., sucrase-isomaltase, lactase, maltase-glucoamylase, α-glucosidase and alkaline phosphatase, which have a positive influence on nutrient degradation and absorption [46,47]. Oral administration of yeast after partial resection of the small bowel, increased disaccharidase activities and improved the absorption of D-glucose as well as the expression of the sodium/glucose cotransporter-1 (SGLT-1) in the brush border of the remaining intestinal segments [48]. Improvement of expression of SGLT-1 by *S. cerevisiae* var. *boulardii*, which is implicated in water and electrolyte re-absorption, could be beneficial in the treatment of diarrhoea and congenital sucrase-isomaltase deficiency. *S. cerevisiae* var. *boulardii* cells contain high level of polyamines and it has been suggested that endoluminal release of polyamines (mainly spermine and spermidin) by *S. cerevisiae* var. *boulardii*, may contribute to rise in expression of intestinal enzymes, *i.e.*, increase in sucrase and maltase activity [49]. 

Modification of luminal short-chain fatty acids (SCFAs) concentration is another trophic effect of the yeast. SCFAs are among the most important metabolites produced by anaerobic bacteria in the colon and are involved in water and electrolyte absorption by the colonic mucosa [50]. Patients on long-term total enteral nutrition have a decrease in number of fecal anaerobic bacteria and in the level of fecal SCFAs [51]. Schneider *et al.* [52] have shown that administration of *S. cerevisiae* var. *boulardii* in these patients increased the level of total fecal SCFAs up to 9 days after termination of the treatment. However, yeast did not modify the fecal flora. This increase in fecal SCFAs concentration may explain the preventive effects of the yeast in enteral nutrition-induced diarrhoea. 

*S. cerevisiae* var. *boulardii* further has the ability to prevent reactions to food antigens. In neonates and young infants, the quality of endoluminal proteolysis is very important in the absorption of completely or incompletely degraded proteins and antigens by the mucosal barrier with increased permeability. This is one of the fundamental mechanisms involved in food protein intolerance. Buts *et al.* [53] have shown the endoluminal release of a leucine aminopeptidase by *S. cerevisiae* var. *boulardii* in rats and thereby enhancement of N-terminal hydrolysis of oligopeptides in both endoluminal fluid and intestinal mucosa. Thus, they proposed that this function of *S. cerevisiae* var. *boulardii* could be important in preventing reactions to food antigens when mucosal permeability is increased. 

### 2.3. Application of *S. cerevisiae* var. *boulardii* in Clinical Trails

*S. cerevisiae* var. *boulardii* has been used in different clinical trails against different diarrhoeal diseases and has shown promising results. Treatment with *S. cerevisiae* var. *boulardii* is well tolerated, except for sporadic reports of fungemia, in immune-compromised patients or patients with severe general or intestinal diseases in most cases infected through an indwelling central venous catheter [54,55,56]. One of the benefits of using *S. cerevisiae* var. *boulardii* as a probiotic is the natural resistance of that to antibacterial antibiotics, thus it can be prescribed to patients receiving antibacterial antibiotic therapy. 

Antibiotic-associated diarrhoea (AAD) is a common complication of treatment with antibiotics caused by disruption of normal gut microbiota and colonization of pathogenic bacteria which results in an acute inflammation of the intestinal mucosa. The most common opportunistic pathogen related to AAD is *C.* *difficile* [57,58,59]. Among other infectious organisms *Staphylococcus aureus*, *Clostridium perfringens*, *Klebsiella oxytoca*, *Candida* species, *E. coli* and *Salmonella* species can be mentioned [60,61]. *S. cerevisiae* var. *boulardii* has been comprehensively evaluated for the prevention of AAD and the potential effect of the yeast in decreasing the ADD in adults and children has been proven [62,63].

Traveller’s diarrhoea is a common health complaint among persons travelling from low risk regions to developing countries where enteric infection is hyper-endemic. Enterotoxigenic *E. coli, Shigella* and *Salmonella* account for about 80% of the cases with an identified pathogen [64]. In a meta-analysis study performed by McFarland [65], it has been concluded that *S. cerevisiae* var. *boulardii* has a significant efficacy on the prevention of Traveller’s diarrhoea.

Several randomized placebo-controlled studies have proven the efficacy of *S. cerevisiae* var. *boulardii* in the treatment and prevention of acute infectious [66,67]. Intestinal disorder and diarrhoea are also common complications in critically ill patients with enteral nutrition which is caused by alteration in the colonic microbiota [51]. The effect of *S. cerevisiae* var. *boulardii* to prevent and reduce the incidence of diarrhoea and to decrease the length of this disease has been demostrated [68]. In the patient with AIDS-associated diarrhoea, the efficacy of *S. cerevisiae* var. *boulardii* has been proven by a randomized, double-blind trail [69,70].

*S. cerevisiae* var. *boulardii* has also shown positive results in patients with irritable bowel syndrome (IBS). In a double-blind, placebo-controlled study, performed on patients with diarrhoea-predominant IBS, administration of *S. cerevisiae* var. *boulardii* decreased the daily number of stools and improved the consistency of the stools [71]. A double-blind study on the patients with Crohn's disease with moderate activity showed that the addition of *S. cerevisiae* var. *boulardii* to conventional therapy considerably reduced bowel movements [72]. In patients with Crohn's disease of the ileum or colon who had been in remission for more than 3 months, treatment with *S. cerevisiae* var. *boulardii* together with the conventional therapy was more efficient in preventing relapse, compared to conventional therapy alone [73]. In patients with mild-to-moderate ulcerative colitis, addition of yeast to the conventional therapy resulted in clinical remission for 68% of patients [74]. In a randomized-placebo study on the patients with Crohn’s disease in remission, addition of *S. cerevisiae* var. *boulardii* to the baseline medications improved intestinal permeability with a decrease in the lactulose/mannitol ratio [75].

## 3. Beneficial Effects of Yeasts on Bioavailability of Nutrients

### 3.1. Biodegradation of Phytate by Yeasts

#### 3.1.1. Antinutritional effects of phytate

Phytic acid or phytate (*myo*-inositol hexakisphosphate, IP_6_) is the primary storage form of phosphorus in mature seeds of plants and it is particularly abundant in many cereal grains, oilseeds, legumes, flours and brans. Phytate has a strong chelating capacity and forms insoluble complexes with divalent minerals of nutritional importance such as iron, zink, calcium and magnesium [76,77,78]. Human as well as monogastric animals like poultry and pigs, lack the required enzymes in the gastrointestinal tract for degradation and dephosphorylation of the phytate complex. Besides, lowering the bioavailability of divalent ions, phytate may have negative influence on the functional and nutritional properties of proteins such as digesting enzymes [79]. In addition, lower inositol phosphates attained from degradation of phytate have a positive role in cancer prevention and treatment [80,81]. 

Dephosphorylation of phytate is catalyzed by phytases (myo-inositol-hexakisphosphate 6-phosphohydrolases). Characterized phytases are nonspecific phosphatase enzymes, which release free inorganic phosphate (P_i_) and inositol phosphate esters with a lower number of phosphate groups. Organisms such as plants and microorganisms extensively produce phytase enzymes and make the minerals and phosphorus present in the phytates available through a stepwise phytate hydrolysis [82]. In food processing, degradation of phytate can be catalyzed either by endogenous enzymes, naturally present in cereals, or by microbial enzymes produced by e.g., yeasts or/and lactic acid bacteria naturally present in flour or added as starter cultures [83]. Accordingly, improved adsorption of iron, zinc, magnesium and phosphorus can be achieved by degradation of phytate during food processing [84,85] or by degradation of phytate in the intestine [86]. 

#### 3.1.2. Phytase activity by yeasts

Phytases are widespread in various microorganisms including filamentous fungi, Gram-positive and Gram-negative bacteria and yeasts [87]. Among yeasts, *Candida krusei* (*Issatchenkia orientalis*) [88], *Schwanniomyces castellii* [89], *Debaryomyces castellii* [90], *Arxula adeninivorans* [91,92], *Pichia anomala* [92,93], *Pichia rhodanensis, Pichia spartinae* [94], *Cryptococcus laurentii* [95], *Rhodotorula gracilis* [96], *S. cerevisiae* [97,98,99,100], *Saccharomyceskluyveri, Torulaspora delbrueckii, Candida spp.* and *Kluyveromyces lactis* [94] have been identified as phytase producers. In a study by Olstorpe *et al.* [92] on the ability of different yeast strains (122 strains from 61 species) to utilize phytic acid as sole phosphorus source, strains of A. adeninivorans and P. anomala showed the highest volumetric phytase activities.

Production of phytase by *S. cerevisiae* has been investigated in different studies [83,99]. The phytase activity of *S. cerevisiae* is partly due to the activity of the secretory acid phosphatases (SAPs), which are secreted by the cells to the growth media and are repressed by inorganic phosphate (P_i_) [99]. However, the phytase activity of yeasts, e.g., during bread leavening, is relatively low [83,101,102]. This could be due to the repression of the SAPs by P_i_[99]. Besides, repression of phytate-degrading enzymes is dependent on the pH and the medium composition. Andlid *et al.* [99] have shown that repression of phytate-degrading enzymes is weak in complex medium with pH 6.0 and high amount of phosphate. Regardless of P_i_ addition, the capacity to degrade phytase is highest at the pH far from the optimum pH for the SAPs, suggesting that pH has more effect on the expression of the enzyme that on the enzyme activity.

*S. cerevisiae* as a phytase carrier in the gastrointestinal tract and hydrolysis of phytate after digestion has also been investigated. In a study using a high-phytase producing recombinant yeast strain at simulated digestive conditions, a strong reduction of phytate (up to 60%) in the early gastric phase was observed as compared to no degradation by wild-type strains. The phytase activity during digestion was influenced by the type of yeast strain, cell density, and phytate concentration. However, degradation in the late gastric and early intestinal phases was insignificant, in spite of high phytate solubility, high resistance against proteolysis by pepsin, and high cell survival [103]. This study also showed the importance of pH as a limiting factor for phytase expression and/or activity, as observed by Andlid *et al.* [99].

#### 3.1.3. Application of yeast phytases in foods

Yeasts or yeast phytases can be applied for pre-treatment of foods to reduce the phytate contents or they can be utilized as food supplement in order to hydrolysis the phytate after digestion. The phytase activity of yeast during bread making for reduction of phytate content of bread have been examined. However, it seems to be too low to significantly influence the iron absorption [99]. Nevertheless, as explained earlier, during bread making, the content of phytic acid decreases. This is due to the action of phytases in the dough (cereal) and the activity of starter culture [83,104,105,106]. Chaoui *et al.* [106] have shown that phytase activity in sourdough bread is highest using combinations of yeasts and lactic acid bacteria as starter culture. The same result was found by Lopez *et al.* [105]. They found that phytate contents in yeast and sourdough bread were lower than in reconstituted whole-wheat flour and that mineral bioavailability could be improved by bread making especially using both yeast and lactic acid bacteria. Therefore a high-phytase S. *cerevisiae* strain, may be suitable for the production of food-grade phytase and for direct use in food production [98]. Increasing the bioavailability of minerals is especially of importance in low-income countries. Therefore it is important to notice that apart from bread, reduction of phytates by yeast phytases have been observed in other plant-derived foods such as in ‘*Icacina mannii* paste’, a traditional food in Senegal, during fermentation with *S. cerevisiae* [107] and in ‘Tarhana’, a traditional Turkish fermented food, using baker's yeast as a phytase source [108]. 

### 3.2. Folate Biofortification by Yeasts

#### 3.2.1. Importance of folate in the human diet

Folates (vitamin B_9_) are essential cofactors in the biosynthesis of nucleotides and therefore crucial for cellular replication and growth [109,110]. Plants, yeast and some bacterial species contain the folate biosynthesis pathway and produce natural folates, but mammals lack the ability to synthesize folate and they are therefore dependent on sufficient intake from the diet [111]. During the last years, folates have drawn much attention due to the various beneficial health effects following an increased intake. The role of folate in the prevention of neural tube defects in the foetus has been established [112,113] and sufficient folate intake may reduce the risk of cardiovascular disease [112,114], cancer [112,115] and even Alzheimer's disease [116]. The recommended dietary intake (RDI) for the adult population is between 200-300 µg/day for males and between 170-300 µg/day for females according to the FAO/WHO in the USA and several European countries [117]. Insufficient folate levels result in prolonged cell division, which leads to megaloblastic anaemia [118]. 

#### 3.2.2. Folate production by yeasts

*S. cerevisiae* is a rich dietary source of native folate and produces high levels of folate per weight [119]. Besides the role as a biofortificant in fermented foods, high producing strains may be used as biocatalysts for biotechnological production of natural folates. The folate level can be considerably augmented in fermented foods using an appropriate yeast strain and by optimizing the growth phase and cultivation conditions for the selected strain. Hjortmo *et al.* [120] have found that the growth medium and physiological state of cells are important factors in folate production. In synthetic growth medium, high growth rate subsequent to respiro-fermentative growth resulted in the highest specific folate content (folate per unit biomass). In complex media, the level of folate was much lower and less related to growth phase. The specific content of folate in yeast is not only species-specific but also dependents on the yeast strain. In another study, Hjortmo *et al.* [121] investigated the folate content and composition and the dominating forms of folate found in 44 different strains of yeasts belonging to 13 different yeast species cultivated in a synthetic medium at standard conditions. There was a large diversity in relative amounts of folate content among the studied yeasts. Tetrahydrofolate (H_4_folate) and 5-methyl-tetrahydrofolate (5-CH_3_-H_4_folate) were the dominating forms, which were varying extensively in relative amounts between different strains. Several strains showed a 2-fold or higher folate content as compared to the control strain, *i.e.,* a commercial strain of Baker's yeast. This indicates that by choosing an appropriate strain, the folate content in yeast-fermented foods may be enhanced more than 2-fold. These scientists have shown that using a specific strain of *S. cerevisiae* cultured in defined medium and harvested in the respiro-fermentative phase of growth prior to dough preparation the folate content increased 3 to 5-fold (135-139 µg/100 g dry matter) in white wheat bread, compared to white wheat bread industrially processed with commercial *S. cerevisiae* (27-43 µg/100 g dry matter) [122].

#### 3.2.3. Effect of yeasts on folate biofortification of food

Cereals, especially whole grain products, are the main supplier of folate in the diet. Yeast has crucial effects on the folate contents of breads. Breads prepared with baking powder have the lowest folate contents, while addition of yeast results in higher folate content in bread [123]. The variety of sourdoughs and baking processes obviously lead to great variation in folate content of breads. Total folate content increases considerably during sourdough fermentation due to the growth of yeasts [123,124]. However, there would be some losses (about 25%) in the amount of folate following the baking [123]. Final folate content is dependent on the microflora and amylolytic activity of flour, starter cultures and baking conditions [125]. Other microorganisms present in the sourdough like lactic acid bacteria may also influence the folate content. In a study, Kariluoto *et al.* [126] investigated the ability of typical sourdough yeasts (*S. cerevisiae*, *Candida milleri*, and *T. delbrueckii*) and lactic acid bacteria to produce or consume folates during sourdough fermentation. Yeasts increased the folate contents of sterilised rye flour-water mixtures to about 3-fold after 19 h, whereas lactobacilli not only did not produce folates but also decreased it to ultimately half amount. Although the lactobacilli consumed folates, their effect on folate contents in co-cultivations with yeasts was minimal.

In beer, the amount of folate enhances due to synthesis by the yeast during the initial period of the fermentation. However, since yeast folate is intracellular, after cropping the yeast, folate will be eliminated from the beer and this is regardless the type of yeast. Some beer brands, which have a secondary fermentation step (often in the bottle), contain higher level of folate [125].

Production of folate in kefir has also been investigated. Kefir is a fermented milk beverage that originated in Eastern Europe and regarded as a natural probiotic product, *i.e.,* a health promoting product [127]. It is produced by the fermentation of milk with kefir granules (grains) and contains different vitamins and minerals. Kefir granules have a varying and complex microbial composition including species of lactic acid bacteria (as the largest portion of microorganism), acetic acid bacteria, yeasts and mycelial fungi. Yeasts isolated from Kefir grains include *Kluyveromyces marxianus*, *Saccharomyces exiguus*, *Candida lambica* and *C. krusei* (*I. orientalis*) [128]. Kefir contains high folate content, which is produced by the yeast and not the lactic acid bacteria [129]. In a study, Patring *et al.* [129] investigated the folate content of different yeast strains isolated from Russian kefir granules, belonging to different *Saccharomyces* and *Candida* species. Kefir yeast strains showed high folate-producing capacity. The most abundant folate forms were 5-CH_3_-H_4_folate (43-59%) and 5-formyltetrahydrofolate (5-HCO-H4folate, 23-38%), whereas H4folate occurred in a minor proportion (19-23%). By choosing yeast strains that produce a higher proportion of the most stable folate forms such as 5-HCO-H4folate and 5-CH3-H4folate, it is possible to improve the stability of folates during fermentation and storage, and thus to increase the folate content in kefir products.

Recently, the folate content of a traditionally fermented maize-based porridge, called togwa, consumed in rural areas in Tanzania has been investigated by Hjortmo *et al.* [130]. The yeasts strains belonged to *C. krusei* (*I. orientalis*)*, P. anomala, S. cerevisiae, K. marxianus* and *Candida glabrata*. The major folate forms found during the fermentations were 5-CH3-H4folate and H4folate. The content of H4folate, per unit togwa, remained quite stable at a low level throughout the experiment for all strains, while the concentration of 5-CH3-H4folate was highly strain- and time-dependent. The highest folate concentration was found after 46 h of fermentation with *C. glabrata*, corresponding to a 23-fold increase compared with unfermented togwa. As for degradation of phytate, selection of appropriate yeast strains as starter cultures in indigenous fermented foods appears to have high potential in especially developing countries where the vitamin intake generally is lower. Compared to e.g., lactic acid bacteria yeast are much more robust and may therefore more easily be distributed as starter cultures.

## 4. Beneficial Effects of Yeasts on Detoxification of Mycotoxins

### 4.1. Prevention of Toxic Effects of Mycotoxins

Mycotoxins are secondary metabolites produced by fungi belonging mainly to the *Aspergillus, Penicillium* and *Fusarium* genera. Agricultural products, food and animal feeds can be contaminated by these toxins and lead to various diseases in humans and livestocks [131]. Contamination of agricultural products by mycotoxins is a worldwide dilemma, however it is rigorous in tropical and subtropical regions [132]. The most important mycotoxins are the aflatoxins, ochratoxins, fumonisins, deoxynivalenol (DON), zearalenone (ZEA) and trichothecenes [133,134]. There are three general strategies in order to prevent the toxic effects of mycotoxins in foods: (i) prevention of mycotoxin contamination (ii) decontamination/detoxification of foods contaminated with mycotoxins and (iii) inhibition of absorption of consumed mycotoxin in the gastrointestinal tract [135]. The ideal solution to reduce the health risk of mycotoxins is to prevent contamination of foods with them. Unfortunately, this can not be completely avoided and sporadically mycotoxin contamination is reported in food products, especially in the developing world [136]. Therefore, there is an increased focus on effective methods for detoxification of mycotoxins present in foods and also on the inhibition of mycotoxin absorption in the gastrointestinal tract. Various physical and chemical methods are available for the detoxification of food products contaminated with mycotoxins. However, due to disadvantages of these methods, such as possible losses in the nutritional quality of treated commodities, limited efficacy, reduction of sensory quality and high cost of equipment, their application has been restricted [135]. An alternative strategy could be utilization of microorganisms capable of detoxifying mycotoxins in contaminated foods and feeds.

### 4.2. Biodegradation of Mycotoxins by Yeasts

Interests in biodegradation of mycotoxins have been increased significantly, since it is specific and environmentally friendly to reduce or eliminate the possible contaminations of mycotoxins in foods. Various microorganisms such as soil or water bacteria, fungi, and protozoa as well as specific enzymes isolated from microbial systems are able to some extent and with varied efficiency to degrade mycotoxins to less- or non-toxic products [137]. Degradation of mycotoxins subsequent to yeast fermentation has been reported in different studies. Degradation of patulin during fermentation of apple juice by *S. cerevisiae* with E-ascladiol and Z-ascladiol as major metabolites [138] and degredation of zearalenone by several yeast strains has been observed [139]. However, degradation of zearalenone leads to conversion of that to α- and β-zearalenol, which are still toxic. Degradation of ochratoxin A, fumonisins B_1_ and B_2_[140], deoxynivalenol and T-2 toxin [141] by *S. cerevisiae* has been reported. Two yeast strains, *Phaffia rhodozyma* and *Xanthophyllomyces dendrorhous,* have also been shown to have ochratoxin A (OTA) degrading activity by converting OTA to ochratoxin α possibly mediated by an enzyme related to carboxypeptidases [142]. 

### 4.3. Mycotoxin Absorption by Yeasts

Inhibition of mycotoxin absorption in the gastrointestinal tract is another way to prevent the toxic effects of mycotoxins. There has been increased interest in the use of mycotoxin binding agents, e.g., yeasts and yeast-derived products, which can be added to the diet to bind mycotoxins. *S. cerevisiae* has the ability to bind mycotoxins as reviewed by Shetty and Jespersen [143]. The mechanism of detoxification by yeast is due to the adhesion of mycotoxins to cell-wall components. As for binding of pathogenic bacteria [16], mannan components of the cell wall play a major role in mycotoxin binding [144]. *In vitro* efficacy of esterified glucomannan to bind aflatoxin B_1_, ochratoxin A and T-2 toxin, when present alone or in combination, was assessed in toxin-contaminated feed. Esterified glucomannan showed significantly higher binding ability to aflatoxin B_1_ than to ochratoxin A and T-2 toxin in a dose dependent manner [145]. In a study by Aravind *et al.* [146] performed on broiler chicks to determine the efficacy of esterified glucomannan in counteracting the toxic effects of mycotoxins in naturally contaminated diet (aflatoxin, ochratoxin, zearalenone and T-2 toxin), it was observed that esterified glucomannan effectively improved the growth depression caused by mycotoxins. Strains of *S. cerevisiae* have been shown to bind ochratoxin A [147] and zearalenone as well [148]. Ochratoxin A and T-2 toxins also bind to glucomannan component of cell wall [145,149]. However, zearalenone bind to β-d-glucans of yeast cell wall [148]. It has been shown that yeast cell wall derived products efficiently adsorbed zearalenone (>70%) in an *in vitro* model that resembled the different pH conditions in the pig gastrointestinal tract, but they were not able to bind deoxynivalenol in a considerable percentage [150]. In study on mice, when dried yeast and yeast cell walls were added to the diet along with aflatoxin B_1_, a significant reduction in the toxicity was observed [151]. Similarly, Madrigal-Santillán *et al.* [152] described the potential of *S. cerevisiae* to improve weight gain and reduce genotoxicity of aflatoxin B_1_ in mice fed with contaminated corn. 

Even though several trials have been made for decontamination of animal feeds by yeast, very little have so far been conducted on decontamination of foods and beverages. Binding of mycotoxins to yeast has especially been investigated during winemaking. It has been shown that yeasts can bind to ochratoxin A and remove it from the white and red wine. Ochratoxin A removal from grape must was due to binding of the toxin to the yeast cell wall, and mannoproteins were involved in the mycotoxin absorption during winemaking. The implication of this finding could be very important in the winemaking of must contaminated with ochratoxin A [153,154]. Oenological strains of *Saccharomyces* yeasts can also be used for the decontamination of ochratoxin A in synthetic and natural grape juice. Heat-treated cells showed higher absorption (90% w/w) compared to viable cells (35% w/w) showing the involvement of physical binding, and cell density played an important role in absorption efficiency. Dead yeasts do not pose any quality or safety problems and can be potentially used for detoxification of the grape juice [147]. *S. cerevisiae* is one of the most important microorganisms involved in food fermentations in tropical countries with high level of mycotoxin contamination in the foods. Shetty *et al.* [155] have investigated Aflatoxin B_1_ binding abilities of *S. cerevisiae* strains isolated from fermented maize dough (Kenkey) and sorghum beer (Pito), indigenous fermented foods from Ghana, West Africa. They showed that aflatoxin binding was strain specific and strains were found to bind 10-40% and some of them more than 40% of the added aflatoxin B_1_ at standard condition. Highest binding capacity was observed at the exponential growth phase with 53% binding of the total toxin and the binding reduced towards the stationary phase. Aflatoxin B_1_ binding increased in a dose dependent manner after addition of aflatoxin, regardless to the temperatures ranging from 20 to 37 °C, but was significantly reduced at 15 °C. Heat and acid treated cells showed higher binding capacity, *i.e.*, up to 78% binding of the total added toxin [155]. Following this study unpublished results by Jespersen *et al.* have shown the yeast-aflatoxin B_1_ complex to be stable during the passage of an *in vitro* gastrointestinal tract model indicating that the aflatoxin will not be absorbed in the gastrointestinal tract but excreted together with the yeast cells in the human feces. Additionally, strains of *S. cerevisiae* isolated from indigenous fermented foods which are effective aflatoxin binders have been proven to be usable as starter cultures with additional capacities to decontaminate mycotoxins in fermented maize products. 

## 5. Conclusions

Yeasts are used in preparation of human foods and beverages, where they besides having technological functions, confer different beneficial effects on human health and well-being. Among these, the most well known is the probiotic effect, which has been proven for *S. cerevisiae* var. *boulardii*. This is the only yeast produced and used as a pharmaceutical product offering numerous valuable effects such as prevention and treatment of intestinal diseases and immunomodulatory effects. Since *S. cerevisiae* var. *boulardii* is recognised as a member of the species *S. cerevisiae,* it is most likely that also other strains within *S. cerevisiae* might show probiotics properties. Even though to date, most efforts have been focused on analysing the probiotic effects of S. cerevisiae var. boulardii isolates. Similarly, other yeast species might have probiotic effects and confer positive effects on human health. Therefore, it is recommended that additional attempts are placed on discovering the probiotic properties among other yeast species and strains. 

Besides the role as probiotics, yeasts have other beneficial functions such as dephosphorylation of phytate and folate biofortification of foods. By choosing appropriate yeast strains as starter cultures and using optimized food processing techniques, it is possible to improve the nutritional value of foods in general. In low-income areas such as the developing countries, application of yeast strains with high phytase and/or folate producing capacity in indigenous food fermentation will increase the bioavailability of minerals and folate content in the foods and will reduce the risk of different diseases such as neural tube defects and anaemia. Therefore, investigation of potential yeast strains isolated from indigenous native foods with high phytase activity and/or high folate content is encouraged.

Contamination of agricultural products by mycotoxins causes severe threat to both livestock productivity and human health and brings huge worldwide economic losses each year. This is especially a rigorous problem in tropical and subtropical regions. For this reason, isolation and application of yeast strains with high mycotoxin binding capacity from indigenous fermented foods would be a valuable alternative for decontamination of mycotoxins in food. Strains of yeast, e.g., *S. cerevisiae* with high mycotoxin binding abilities can be used as part of the starter cultures in the fermentation of food and beverages, and heat treated cell walls or purified components can be applied as additives in small quantities without compromising the characteristics of the final product. The application of *S. cerevisiae* as mycotoxin binders in human foods highly depends on stability of the yeast-mycotoxin complex through the passage of the gastrointestinal tract. Additional research efforts are required in this area to explore the great potential of using *S. cerevisiae* as a detoxifying agent in contaminated foods.

So far, tremendous efforts have been placed on utilising the probiotic effects of especially lactic acid bacteria, whereas rather limited emphasis has been placed on the beneficial effects offered by yeast. Yeasts do meanwhile offer several advantages compared to lactic acid bacteria. They do have a more diverse enzymatic profile, they appear to have a more versatile effect on the immune system, they do provide protection against pathogenic bacteria and toxic compounds by surface binding and appear to be better suited for nutritional enrichment and delivery of bio-active molecules. Besides yeast are much more robust than lactic acid bacteria which make them easier to produce and to distribute, especially in less developed areas. It is therefore encouraged that additional efforts are placed on exploring the health beneficial effects of yeasts, especially those properties that can not be replaced by lactic acid bacteria.
